# Vertical root fractures: A time-dependent clinical condition. A case-control study in two colombian populations

**DOI:** 10.4317/jced.58701

**Published:** 2021-11-01

**Authors:** Claudia García-Guerrero, William Mendoza-Beltrán, Mateo Roldan-Roldan, Paula Villa-Machado, Felipe Restrepo-Restrepo

**Affiliations:** 1 DDS, MSc. Universidad Nacional de Colombia, Sede Bogotá, Facultad de Odontología, Departamento de Ciencias Básicas y Medicina Oral, Grupo de investigación INVENDO, Bogotá D.C., Colombia; 2 DDS. Universidad Nacional de Colombia, Sede Bogotá, Facultad de Odontología, Departamento de Ciencias Básicas y Medicina Oral, Grupo de investigación INVENDO, Bogotá D.C., Colombia; 3 DDS. Endodontist, Universidad de Antioquia. Medellín, Colombia, Laboratory of Immunodetection and Bioanalysis. Medellín, ColombiaDDS. Endodontist, Universidad de Antioquia. Medellín, Colombia, Laboratory of Immunodetection and Bioanalysis. Medellín, Colombia; 4 DDS. Endodontist, Universidad de Antioquia. Medellín, Colombia, Faculty of Dentistry, Laboratory of Immunodetection and Bioanalysis. Medellín, Colombia

## Abstract

**Background:**

This nested case-control study can be viewed as an efficient way to sample subjects from a large cohort study case-control study aimed to analyze the effect of different clinical factors on the appearance of vertical root fractures in endodontically-treated teeth (ETT) over time.

**Material and Methods:**

By matching 90 cases and 270 controls nested in a cohort of 450 patients. Incident “cases” included those ETT in which a confirmed VRF. The “controls” were ETT with clinical and radiographic evidence of normality. When an “incident case” was detected, three random “controls” according to the evaluation time registered in years were selected. Time interval corresponded to the exposure time from the end of the endodontic treatment until the tooth was included in the study. Demographic and clinical parameters included: age, gender, type, and location of the tooth, type of endodontic treatment, number of appointments necessary to complete the endodontic treatment, use of intra-canal medication, the apical extension of the filling, type of coronal restoration, the role of the tooth in the rehabilitation treatment, presence of intra-radicular posts, and presence of an adjacent implant, were analyzed over time. Statistical analysis: univariate descriptive analysis, Pearson’s χ2 test, and a logistic regression model adjusted for the most significant variables with a 95% confidence interval.

**Results:**

The prevalence of vertical root fractures was 16.42%. The multivariate analysis confirmed that re-treatment (OR:12.19; OR:4.28;*P*<0.05) lasting five to ten years and intra-canal medication (OR:6.16;*P*=0.004) for more than eleven years significantly more associated with the risk of vertical root fracture. For teeth with intra-canal post or direct coronal restorations, the risk of vertical root fracture was three times lower.

**Conclusions:**

Endodontic re-treatment and the use of intracanal medication such as calcium hydroxide should be considered primary and secondary risk factors, respectively, according to the appearance of VRF over time.

** Key words:**Apical surgery, endodontic re-treatment, endodontically-treated teeth, risk factors, vertical root fracture.

## Introduction

Vertical root fracture (VRF) is defined as a complete or incomplete longitudinal break that extends along the vertical axis of the tooth root (1), and it is considered one of the most common causes of dental extraction (2). The prevalence of VRF is between 4.3% and 13.4% (2,3), and patient-specific etiological factors have been associated with the occurrence of VRF, including root morphology, type of tooth, location in the arch, and physiological changes of the dental tissue due to senescence (4), or dentinal cracks (2). *In vitro* studies have not consistently found a direct correlation between mechanical instrumentation and the appearance of cracks in the root canal dentin (2,5); however, clinical evidence supports an association between the presence of VRF and previous endodontic treatment (3).

Regarding the biological mechanisms behind VRF on endodontically-treated teeth, it has become more apparent how the loss of mechanoreceptors present in the pulp tissue would reduce the protection required during masticatory impact, causing (in short periods) fracture of the coronal tissue (6). Furthermore, parafunctional habits were significantly associated with the presence of VRF, implying that a change in the proprioception mechanism for endodontically-treated teeth could reduce the responsiveness to functional or parafunctional chewing loads (7).

The loss of the protective effect of the pulp tissue during chewing adds to the microstructural changes in the dentin that promote instrumentation; the chemical conditioning (8) and excessive forces during filling (9) are plausible factors that contribute to the failure of the dental structure, especially over time. Therefore, if 66% of VRFs were diagnosed between 2 to 5 years from the completion of root canal treatment (9), the structural changes that the treated root dentin undergoes are considered time-dependent, and the multifactorial nature of the vertical fracture does not allow the identification of a single risk factor (4). Thus, tooth structural loss, viscoelasticity loss, age-induced changes in the dentin, and restorative procedures are primary factors that usually predispose the teeth to immediate fracture. In contrast, secondary causes like chemical effects, bacterial interactions, and biocorrosion predispose the teeth to fracture after some time (4).

Therefore, this study aimed to analyze in two Colombian populations, different patient- and treatment-related factors associated with the time of presentation of VRFs in endodontically-treated teeth.

## Material and Methods

-Study design

Patients attending the Endodontics Postgraduate Clinic at the Faculty of Dentistry of the Universidad Nacional de Colombia (FOUN), Bogotá – Colombia, and the Faculty of Dentistry of the Universidad de Antioquia (FOUA), Medellín – Colombia, were recruited. An observational nested case-control study was carried out, comparing endodontically-treated teeth (ETT) with and without clinical evidence of VRF. The occurrence of VRF (cases) was identified surgically.

The study protocol followed the Strengthening the Reporting of Observational Studies in Epidemiology (STROBE) guidelines for case-control studies (10) and was approved by the Ethics committees of both Universities, following the principles established in the Declaration of Helsinki.

-Inclusion and exclusion criteria

All participants with crowned permanent teeth, complete root formation, and history of endodontic treatments such as root canal treatment, root canal re-treatment, and apical surgery were included. For each patient, a complete endodontic chart, date of end of treatment, and final X-ray were obtained. Patients with current orthodontics treatment, history of trauma, tooth decay, and coronal fractures were excluded.

-Sample size

The sample size was calculated based on a preliminary study with an initial cohort of 143 participants in which the odds ratio (OR) value for the factor “presence of direct restorations” was; OR: 4.95 with a probability of exposure factor of 0.5 and 0.32 for cases and controls, respectively (11). A minimum sample size of 331 ETT containing 83 cases and 248 controls to reach a 95% confidence level, with an alpha value of 5% and a power of 80% in identifying significant differences in the between-group comparisons (12). Likewise, to include the most significant number of subjects, according to the sample size calculation, a consecutive non-probabilistic selection of participants who met the eligibility criteria was established.

-Selection of cases and controls

**Figure 1:**
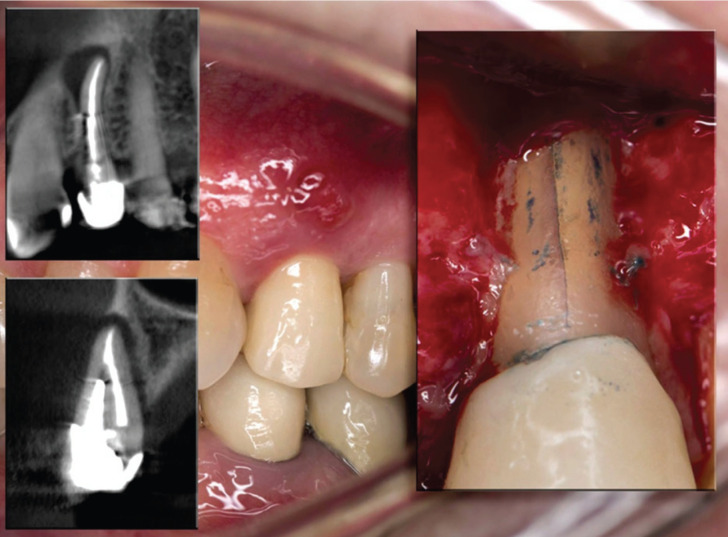
Tomographic image and clinical photograph confirming the finding of VRF.

**Figure 2:**
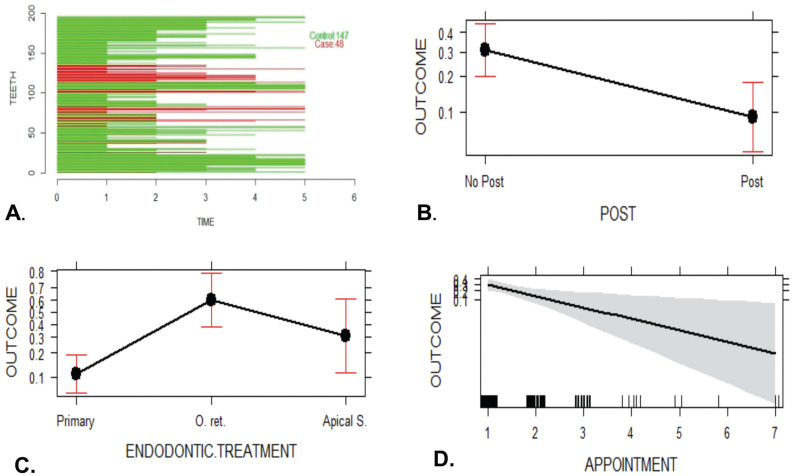
Group 1 (1–5 years); A. Probability analysis of the presence or absence of VRF; B plot effect for variable fracture probability according to presence of post; C plot effect for variable fracture probability according to endodontic treatment; D plot effect for variable fracture probability according to number of appointments.

**Figure 3:**
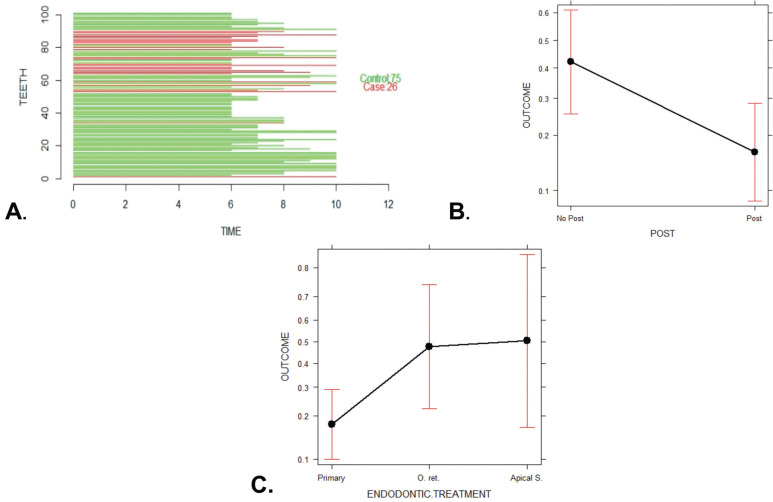
Group 2 (>5–10 years); A. Probability analysis of the presence or absence of VRF; B plot effect for variable fracture probability according to presence of post; C plot effect for variable fracture probability according to endodontic treatment.

**Figure 4:**
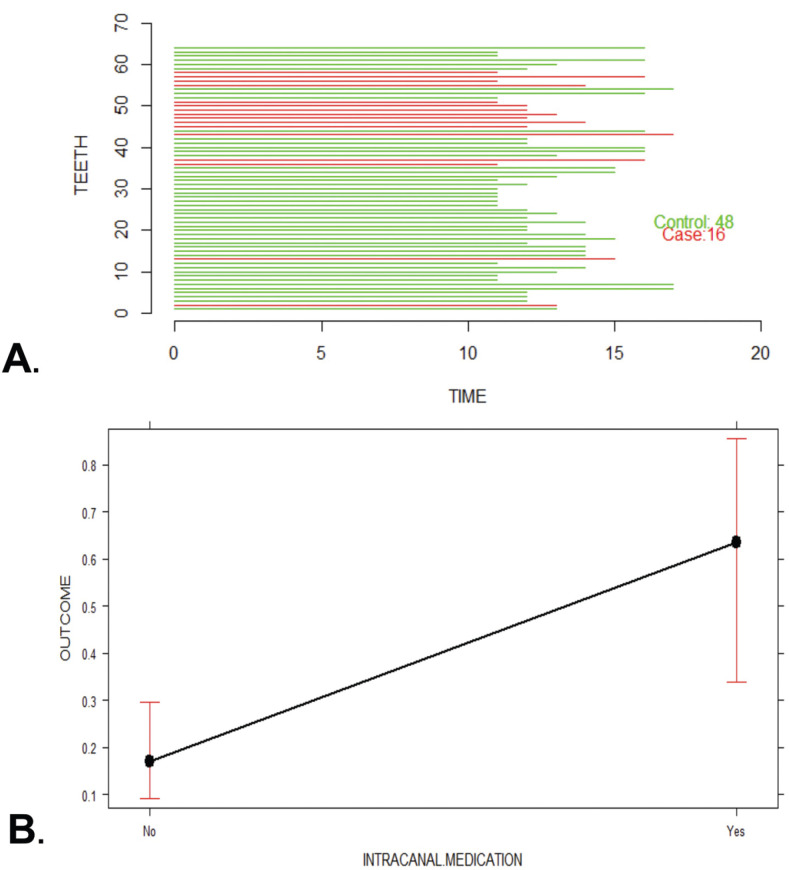
Group 3 (> 10-17 years); A. Probability analysis of the presence or absence of VRF; B plot effect for variable fracture probability according to presence of intra-canal medication.

A total of 481 endodontically treated teeth formed the initial cohort to nest the cases and controls later. The cohort reconstruction was carried out to collect clinical data and radiographic images before and after treatment recorded in the FOUN and FOUA databases up to the year 2020. Incident “cases” included those ETT in which an Endodontist surgically confirmed VRF through surgical flap elevation and direct observation of fracture under a dental operating microscope after presenting any of the following clinical, radiographic or tomographic examinations, supplemented the records per case. signs: localized inflammation, increased mobility, presence of sinus tract adjacent to the gingival margin, angular or lateral bone resorption patterns with lateral extension, and compromised crestal bone height (7) (Fig. 1). The “controls” were ETT with a complete absence of clinical inflammation and radiographic evidence of normality. When an “incident case” was detected, the investigators selected three random “controls” according to the evaluation time registered in years. Time interval corresponded to the exposure time from the end of the endodontic treatment until the tooth was included in the study, this time interval was called as the “etiological window”. Thus, cases were classified into three groups according to the time from ETT was finished to the diagnosis of VRF: Group 1: 1–5 years (Fig. 2A), Group 2: 6–10 years (Fig. 3A), and Group 3: 11–17 years (Fig. 4A), and the cases and controls inclusion into the three groups ensured the random distribution of clinical factors.

-Exposure factors

Demographic and clinical parameters included: age, gender, type, and location of the tooth, type of endodontic treatment, number of appointments necessary to complete the endodontic treatment, use of intra-canal medication (calcium hydroxide), the apical extension of the filling, type of coronal restoration, the role of the tooth in the rehabilitation treatment, presence of intra-radicular posts, and presence of an adjacent implant.

-Statistical analysis

Univariate descriptive analysis for the distribution and frequency of variables in each group was carried out; then variable pairing by the time of exposure was performed. With a 95% confidence level, a bivariate analysis estimated the risk association between the exposure factors and the occurrence of VRF. Pearson’s Chi-square test was used to test the difference between exposure to the risk factor and the presence (or absence) of VRF. A logistic regression model adjusted for the estimated confounding variables, with a 5% significance threshold, the association of risk, and the probability of presenting VRF according to the exposure factors. Data were analyzed using R, version 3.1.3 (http://www.r-project.org/). The threshold for statistical significance was set at *P*<0.05.

## Results

According to the calculation of the sample size, an initial cohort of 481 patients, who had at least one endodontic treatment, allowing the selection of 90 cases and 270 controls, who met the eligibility criteria. The prevalence of VRF in ETT was 16.42%, and the distribution and frequencies of the variables are presented in Table 1,  Table 1 cont.

**Table 1:**
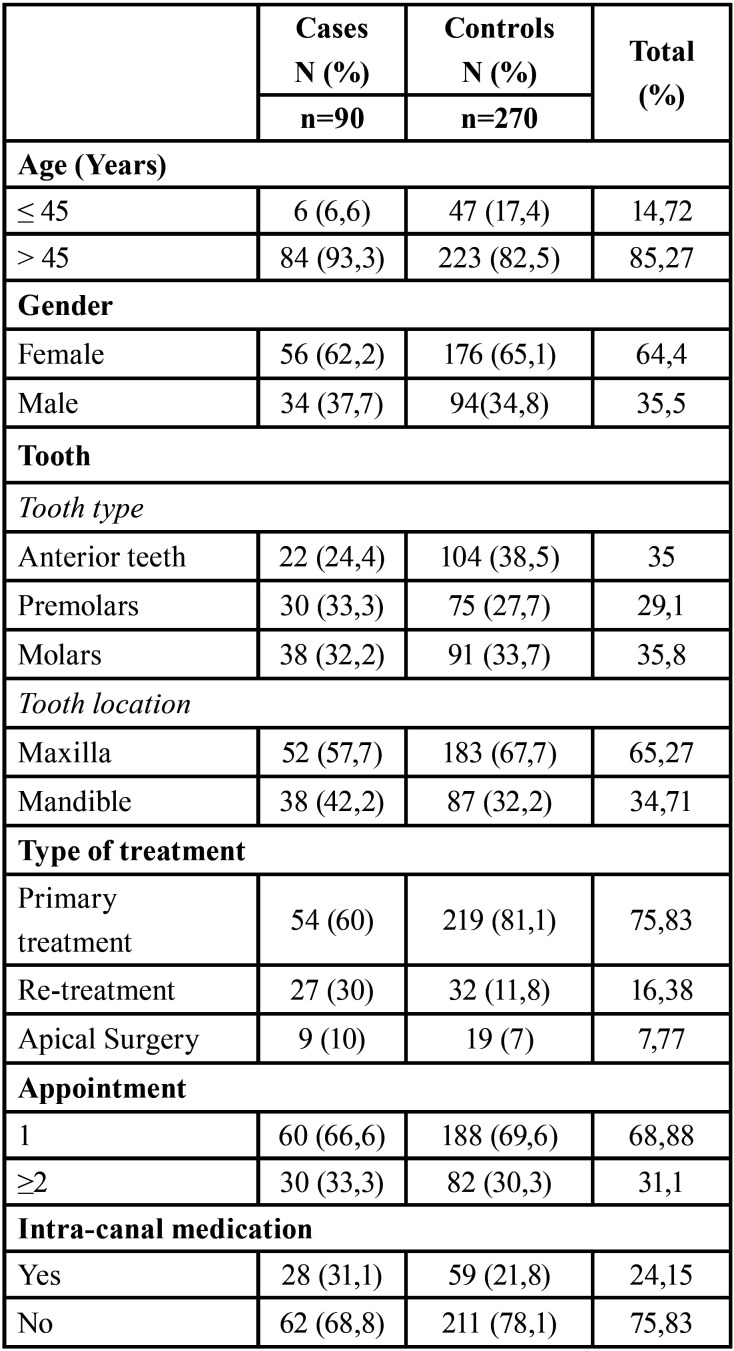
Percentage distribution of the analyzed variables in the study group.

**Table 1 cont.**
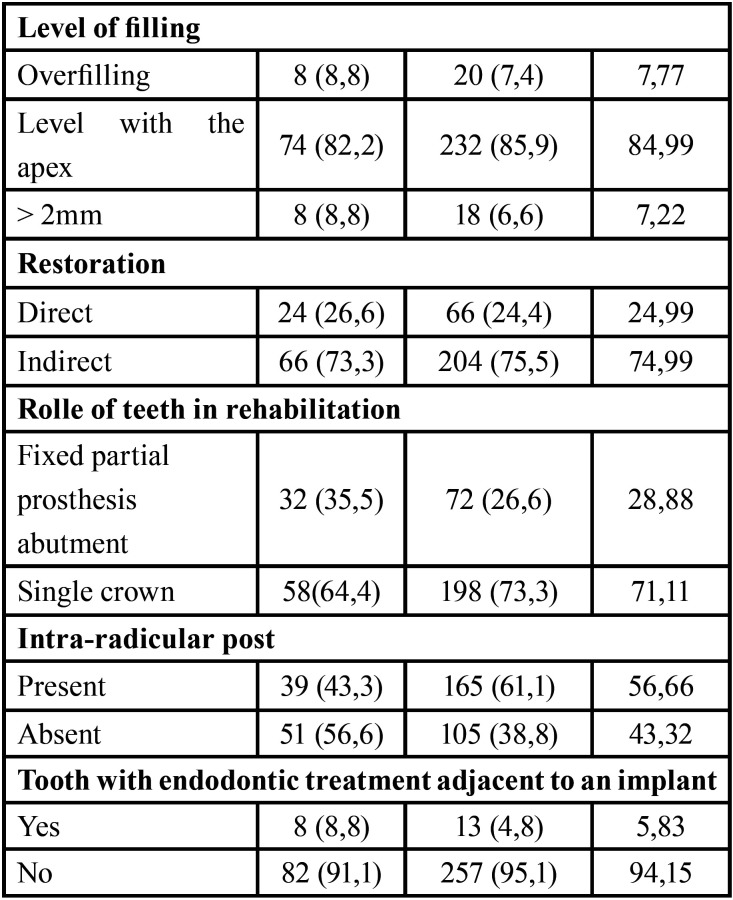
Percentage distribution of the analyzed variables in the study group.

The bivariate analysis showed that the type of endodontic treatment and intra-canal medication had a significant association with the occurrence of VRF. In Groups 1 and 2, a tooth with primary endodontic treatment was three times more likely not to show VRF than one with secondary endodontic treatment, either re-treatment or apical surgery (Group 1: OR: 3.31; 95% CI: 1.65–7.8; *P*=0.003 and Group 2: OR: 3.1; 95% CI: 1.22–10.62; *P*=0.04). Regarding teeth with intra-radicular posts, the probability of presenting VRF decreased (Group 1: OR: 0.42; 95% CI: 0.2–0.82; *P*=0.02). For Group 3, the risk of presenting VRF after the use of intra-canal medication was six times higher (OR: 6.16; 95%IC: 1.99–30.49; *P*=0.004).

The multivariate analysis, previously adjusted for confounding factors, confirmed that the presence of intra-radicular posts in Groups 1 and 2 was identified as a protective factor against VRF (Group 1: OR 0.22, CI, 0.08–0.57, *P*=0.002 and Group 2: OR 0.26, CI, 0.09–0.78, *P*=0.02; Figs. 2B,3B).

 Opposite, endodontic re-treatment presented predisposed significantly more to VRF than primary endodontic treatment in Group 1 (OR 12.19; CI, 3.64–40.81; *P*=<0.0001) as well as in Group 2 (OR 4.28, CI, 1.077–17.07, *P*=0.0388; Fig. 2C,3C). Apical surgery presented a risk trend in Group 1 (OR 3.65, CI, 0.93–14.27, *P*=0.06) and in Group 2 (OR 4.76, CI, 0.80–28.04, *P* = 0.085); however, this result was not statistically significant (Table 2, Figs. 2C,3C).

**Table 2:**
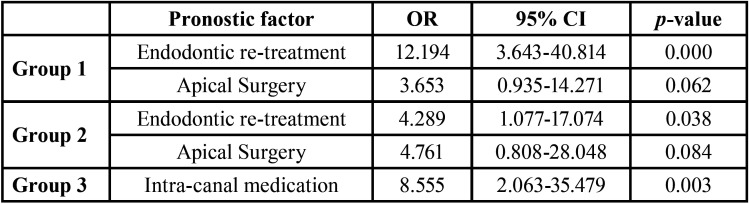
Logistic Regression Analysis.

The effect mediated by the evaluation time for the number of appointments indicated that for teeth from Group 1, the number of appointments was inversely correlated with the appearance of VRF (OR 0.36, 95% CI, 0.17–0.79, *P*=0.01). In contrast, for teeth from Group 3, the use of intra-canal medication was associated with a significant VRF (OR: 6.16, 95% CI: 1.99–30.49, *P*=0.004; Fig. 4B). These findings, explained in terms of probability, showed that according to the type of endodontic treatment, teeth from Group 1 were 10% more likely to present VRF if they had primary endodontic treatment, 30% if they had undergone apical surgery, and 60% if they had endodontic re-treatment. For those from Group 2, the probability of presenting VRF increased by 8% after primary endodontic treatment and 20% after apical surgery. In contrast, the probability of VRF occurring in re-treated teeth was reduced by 12%. Finally, for cases from Group 3, those in which intra-canal medication was used were significantly more likely to suffer VRF (OR 8.55, CI, 2.063–35.47, *P*=0.0031). That is, a 64% increase in the probability of occurrence of VRF was perceived (Fig. 4B).

The etiological window between root filling and the diagnosis of VRF ranged from 1-17 years; the highest incidence (53.3%) was observed between 1 and 5 years (Fig. 2A), Followed by 28.8% of the cases identified between 6 and 10 years, Fig. 3A. Finally, 17.7% of the cases corresponded to treatments of 11 years or more of evolution (Fig. 4A).

## Discussion

VRF is an endodontic complication of multifactorial etiology that leads to tooth loss. This case-control study showed a VRF prevalence of 16.42%, exceeding the 4.3% and 13.4% previously reported in the literature (2,3). A prior standardization for data registration (11), matching by follow-up periods between cases and controls, and assessment of two different populations aimed to overcome the limitations of the case-control study design, at least partially.

As the main finding, the effect that risk factors represented for the occurrence of VRF must be analyzed over time intervals. Thus, an observation period of more than 11 years confirms that intra-canal medication represented a secondary risk for the production of VRF, which could suggest that chemical changes in root dentin are evident over longer observation periods.

Calcium hydroxide has a high pH and antibacterial properties and is highly recommended as an intra-canal medication for the endodontic management of infected teeth between appointments; however, reports in the literature have shown that its prolonged use reduces dentin resistance to fracture by 12.6% (13). Calcium hydroxide has a high pH and antibacterial properties and is highly recommended as an intra-canal medication for the endodontic management of infected teeth between appointments; however, reports in the literature have shown that its prolonged use reduces dentin resistance to fracture by 12.6% (13). Studies have shown that long-term intracanal dressing with calcium hydroxide negatively influences the physical properties of dentin (14). Changes in the organic matrix by the high alkalinity of calcium hydroxide, could dissolve, denature or neutralize acidic organic matrix components, disrupting the bond between collagen fibrils and hydroxyapatite crystals (14). This weakens the tooth structure, leading to accelerated fatigue crack propagation during cyclic stresses and an increase in the susceptibility to root fracture (14). Although according to *in vitro* findings, the chemical-induced effects on the dentin substrate may occur for a more extended time than anticipated the use of irrigation solutions and intracanal medications such as calcium hydroxide could promote long-term damage, thus, it is possible to consider them as secondary risk factors (15,4).

Regarding to, the wear generated during the orthograde re-treatment was considered a primary risk factor (4), as it affected the dental structure between 1 and 5 years. In orthograde re-treatment, the removal of all filling material from the root canals is essential to ensure disinfection; however, this process involves additional mechanical manipulation of the root walls, generating more considerable wear on the tooth structure (8). Shemesh *et al*. found that orthograde re-treatment could generate irreversible damage to the dentin, resulting in cracks and fractures (16). Those results are consistent with those of our study, as it was determined that an endodontically-retreated tooth, followed between one and five years, has an approximately twelve-fold higher VRF risk than other therapeutic alternatives such as primary endodontic treatment or apical surgery. Essential clinical decision-making regarding the re-treatment of initial endodontics treatment observed in normality and function, must be analyzed in the researching evidence (17).

These findings confirm how endodontic treatment impacts radicular dentin, and its role in the occurrence of VRF is shown (18). Liao *et al*. (19) reported that the appearance of VRF in ET is higher (86.15%) than in teeth without previous endodontic treatment (13.85%). The authors agreed on how the endodontic treatment, per se, becomes a determinant for the occurrence of the event, confirming that other factors inherent to the tooth or the patient may not be significant. Karygianni *et al*. observed a higher prevalence of VRF (62.1%) in teeth with orthograde re-treatment and apical surgery. In keeping with this work, a secondary treatment could represent cumulative damage to the root structure and might be a risk factor for VRF (20).

Longitudinal studies at 5 years identify the highest incidence of VRF in the 4.5-year period; however, they exclude conditions such as orthograde re-treatment and the presence of intra-radicular posts, an event that explains the variability observed when trying to identify a risk factor. In light of our results, these conditions are not comparable (21).

The presence of intra-radicular posts has always generated controversy with reference to the greater or lesser susceptibility of the dental structure to fail mechanically (22) *in vitro* studies have identified the intra-radicular post as a potential risk factor for the loss of the ETT, particularly if it was the cast type (22,23). Although these findings could be helpful as a first step. Additional experiments are indicated under clinically realistic and dynamic loading conditions (4).

 In this work, the presence of intra-radicular posts did not determine risk association to VRF in any observation time; in contrast, it was possible to determine how the teeth carrying this type of restoration were significantly more likely to not suffer VRF, in both Group 1 and Group 2. This hypothesis, confirmed by the preliminary study (11), is consistent with the findings of a previous study (22), in which the presence of intra-radicular posts made of fiberglass increased the survival of ET by 4.7%.

Anatomical conditions in posterior teeth, whose flattened roots at mesio-distal suggest two root canals joined by canals or isthmuses, increase the possibility of occurrence of VRF in ETT (24). Reports indicate how the root canal isthmus could act as a natural crack that decreases the force required to resist tooth fracture (25). In the present study, the clinical frequency of VRF was higher in molars (42%) than in premolars (30%), although the difference was not statistically significant. The anatomical complexities require eliminate of the cervical constriction, to reach in straight path at the middle third of the root canal, nevertheless, the increased loss of tooth structure in endodontically treated teeth reduced stability with increased deformation and radicular tensile strain. This alteration in the biomechanical response of root dentin may increase the risk of structural failure with time, and can contribute to the higher prevalence of VRF in non-vital teeth (26). The present work did not discriminate the influence of cervical wear on the appearance of VRF, therefore with the aim of minimally invasive endodontics, more clinical research will be required to explain this mechanism.

Finally, the success of endodontic treatment is conditioned to the healing process (27); however, the permanence of ETT is measured by survival studies (28). According to the results reported by Lee *et al*., 9% of the teeth that underwent primary endodontic treatment were extracted due to the presence of VRF (29); this result coincides with the probability of occurrence recorded in the present study for the primary endodontic treatment (10%). Borén *et al*. analyzed the survival of 420 ETT and showed that 80% of them remained in the mouth after ten years, VRF was the cause of tooth extraction in 9.7% of the cases (30). Therefore, if 53% of cases of VRF occurred in the first five years, an adequate coronal seal will protect the tooth structure and the endodontic filling from masticatory stress and microfiltration. Controlling these factors would increase dental survival by reducing the risk of dental and endodontic treatment failure.

## Conclusions

The risk factors involved in the development of VRF become clinically significant at different time intervals. Therefore, the accumulated wear that promotes orthograde endodontic re-treatment negatively impacts the tooth structure in the first five years; this statement makes it possible to consider endodontic re-treatment as a VRF primary risk factor.

In contrast, the possible chemical alteration that promotes the use of CaOH2 as an intra-canal dressing generates a secondary impact for periods of more than ten years.
